# Improved arthroscopic one-piece excision technique for the treatment of symptomatic discoid medial meniscus

**DOI:** 10.1186/s13018-017-0661-5

**Published:** 2017-10-30

**Authors:** Hong-De Wang, Tong Li, Shi-Jun Gao

**Affiliations:** 1 Department of Orthopedics, The Third Hospital of Hebei Medical University, 139 Ziqiang Road, Shijiazhuang, 050051 Hebei People’s Republic of China; 2 Orthopaedic Biomechanics Laboratory of Hebei Province, 139 Ziqiang Road, Shijiazhuang, 050051 Hebei People’s Republic of China

**Keywords:** Discoid medial meniscus, Improved one-piece excision, Technique

## Abstract

**Background:**

Discoid medial meniscus is an extremely rare abnormality of the knee. During arthroscopic meniscectomy for symptomatic discoid medial meniscus, it is difficult to remove the posterior portion of the meniscus because of the confined working space within the compartment and the obstruction caused by the anterior cruciate ligament and the tibial intercondylar eminence. To overcome these problems, we describe an improved arthroscopic technique for one-piece excision of symptomatic discoid medial meniscus through three unique portals.

**Methods:**

Three improved portals were made in the injured knee: a standard anteromedial portal, a central transpatellar tendon portal, and a high anterolateral portal. The anterior side of the discoid medial meniscus was cut 7 mm from the periphery of the meniscus. Next, the anterior portion of the free discoid meniscus fragment was pulled in the anterolateral direction with tension. A curve-shaped cut was made along the longitudinal tear to the posterior horn using basket forceps through the standard anteromedial portal. Then, the anterior portion of the free discoid meniscus was pulled in the anteromedial direction. Pulling the fragment under tension made it easier to cut the posterior side of the discoid meniscus. The posterior side of the discoid meniscus was cut 7 mm from the periphery of the meniscus with straight scissors or basket forceps through the central transpatellar tendon portal.

**Results:**

This technique resulted in satisfactory results. Excellent visualization of the posterior part of the discoid medial meniscus was gained during the procedure, and it was easy to cut the posterior part of the discoid medial meniscus. No recurrent symptoms were found.

**Conclusions:**

This improved arthroscopic one-piece excision technique for the treatment of symptomatic discoid medial meniscus enables the posterior part of the meniscus to be cut satisfactorily. Moreover, compared with previous techniques, this novel technique causes less formation of foreign bodies and less damage to the anterior cruciate ligament, medial collateral ligament, and cartilage and requires a shorter procedural time.

## Background

The first case of a discoid medial meniscus was reported in 1941 [1]. The discoid medial meniscus is an extremely rare abnormality of the knee, with an estimated incidence of 0.12% [2]; however, the real incidence of discoid medial menisci is difficult to ascertain, as an unknown percentage of discoid medial menisci may be asymptomatic. Many patients with discoid medial meniscal injury are diagnosed based on symptoms such as knee pain, effusion, and locking. Most discoid meniscal injuries cannot be treated non-operatively, as the poor healing capacity results in poor clinical outcomes [3–5]. Therefore, surgical treatment is necessary.

Many surgical techniques have been described for arthroscopic discoid meniscectomy [6–8]. Kim et al. described the partial excision of a symptomatic lateral discoid meniscus in one piece through three portals: the lateral patellofemoral axillary portal, far anteromedial portal, and low anterolateral portal [6]; this technique effectively decreased the risk of the formation of foreign bodies. Subsequently, Ogata described an arthroscopic technique for excision of the complete discoid lateral meniscus with a tear, in which the discoid meniscus is divided into anterior and posterior pieces for removal [8]; this technique can achieve excellent visualization of the posterior segment, which makes it easy to determine the width of the rim that needs to be retained. However, these excellent techniques are concerned with discoid lateral meniscus tear.

Although the discoid medial meniscus is a rare abnormality, we have successfully treated six cases from January 2010 to January 2015. We have found that the surgical treatment for symptomatic discoid medial meniscus differs from discoid lateral meniscus surgery; it is difficult to remove the posterior portion of the medial meniscus because of the confined working space within the compartment and the obstruction caused by the anterior cruciate ligament (ACL) and the tibial intercondylar eminence.

To overcome these problems, we describe an improved arthroscopic technique of the one-piece excision through three unique portals for patients with symptomatic discoid medial meniscus.

## Surgical procedure

**Fig. 1:**
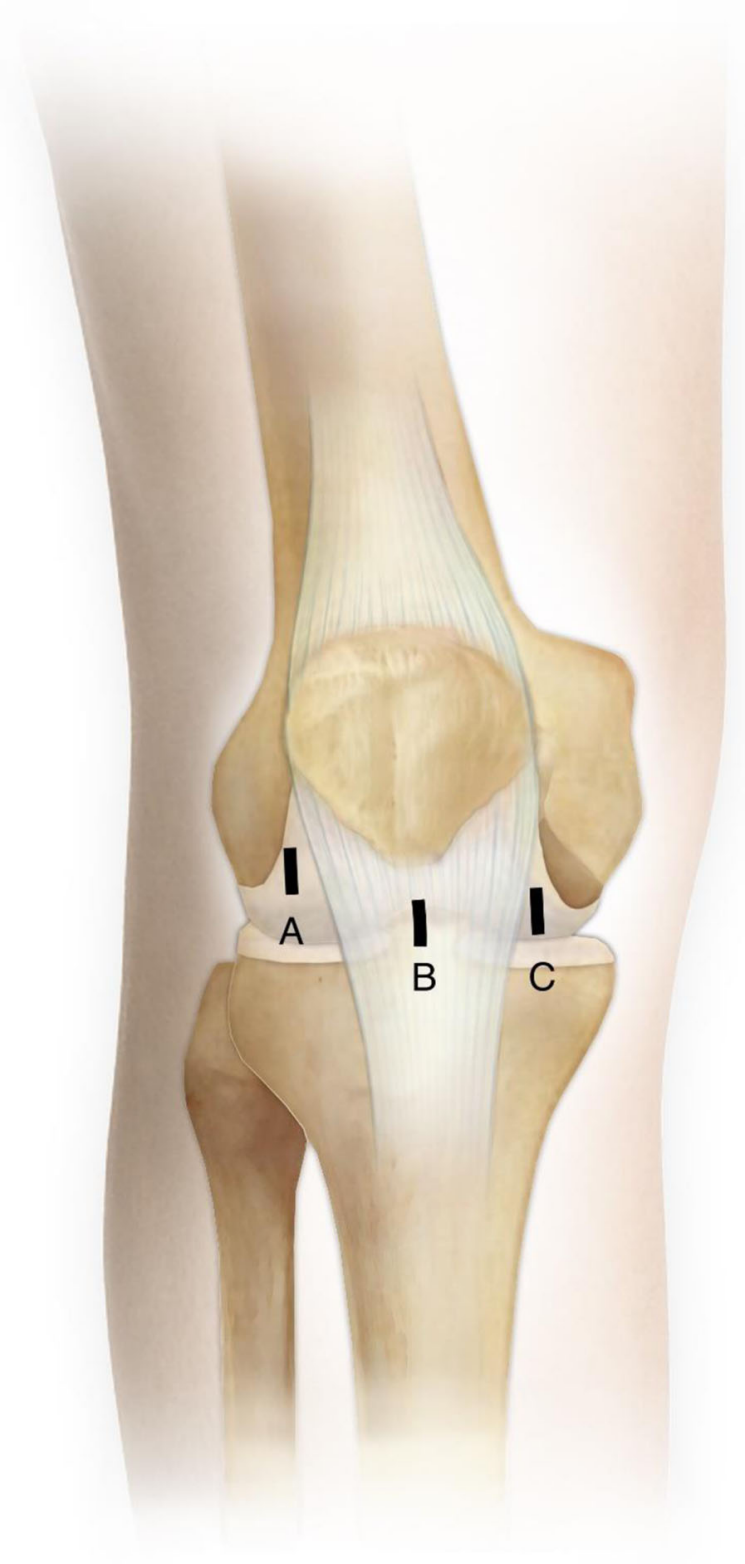
The three portals used in the improved arthroscopic one-piece excision technique for the discoid medial meniscus. A: Standard anteromedial portal, B: central transpatellar tendon portal, C: high anterolateral portal

The extent of the tear in the discoid medial meniscus is confirmed on magnetic resonance imaging to gain an indication of how much of the intact rim can be preserved. The patient is then placed under epidural anesthesia. Three improved portals are made: a standard anteromedial portal, a central transpatellar tendon portal, and a high anterolateral portal (Fig. 1). The standard anteromedial portal is located 1 cm above the medial joint line, 1 cm inferior to the tip of the patella, and 1 cm medial to the edge of the patellar tendon; this is the main working portal, enabling cutting and pulling of the meniscus. The central transpatellar tendon portal is located approximately 1 cm inferior to the lower pole of the patella in the midline of the joint through the patellar tendon; this portal is used for cutting and viewing. The high anterolateral portal is located 1.5 cm above the lateral joint line and 1 cm lateral to the edge of the patellar tendon; this is the main viewing portal.

**Fig. 2:**
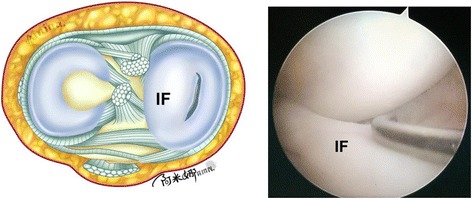
Illustration and intraoperative photograph of a longitudinal tear in the discoid medial meniscus (right knee). The tear in this case example was vertical and extended completely through the thickness of the medial meniscus. The inner fragment protruded over the intercondylar notch. IF inner fragment

**Fig. 3:**
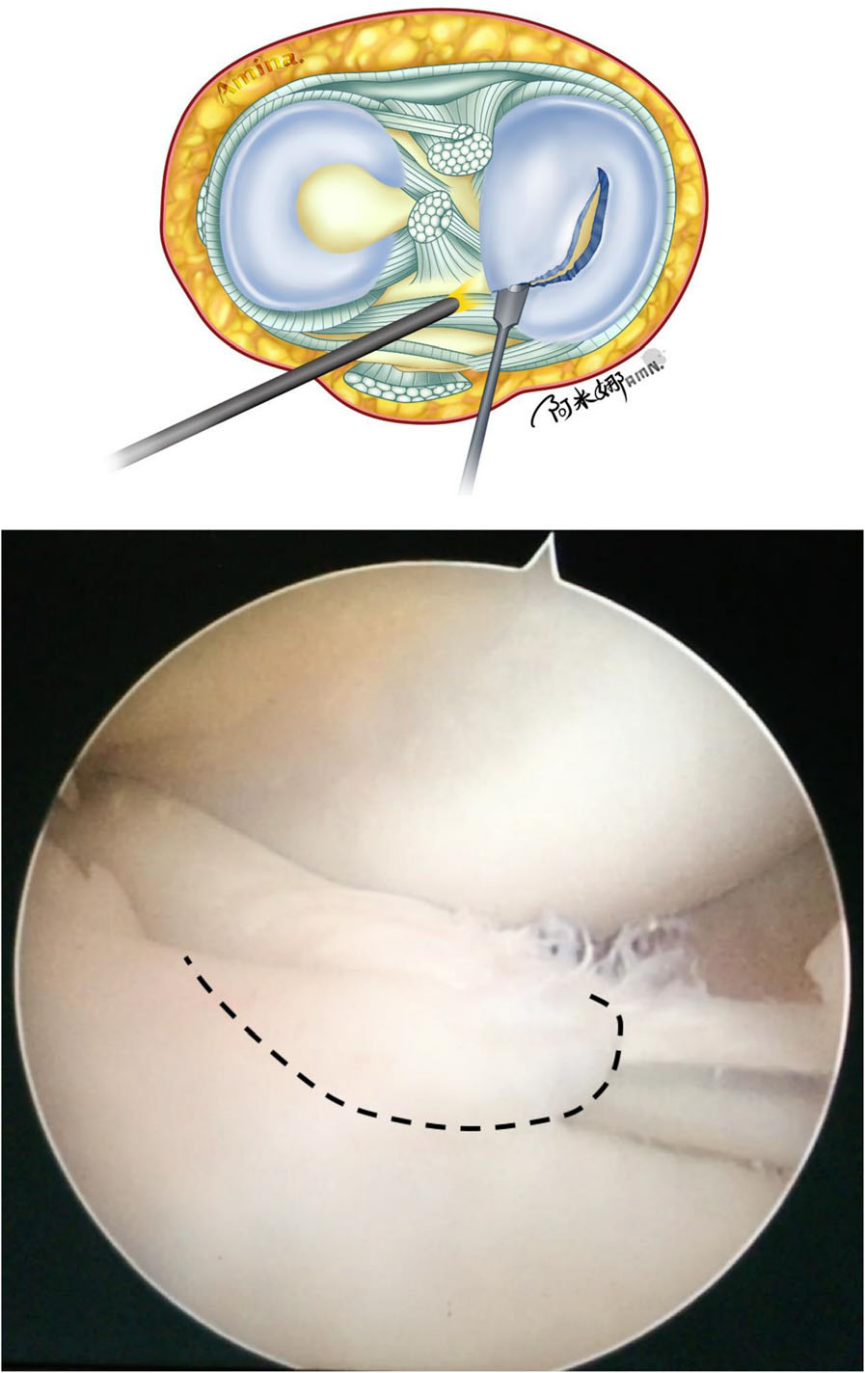
Illustration and intraoperative photograph showing the cut made in the discoid medial meniscus. An arthroscopic knife was carefully inserted through the standard anteromedial portal. The anterior side of the discoid medial meniscus was cut 7 mm from the periphery of the meniscus along the black dotted line

First, a 30° angle arthroscope is inserted through the high anterolateral portal. A probe is then inserted through the standard anteromedial portal, and careful estimation is done to determine the proper peripheral extent of the remaining meniscus under valgus stress. In the case example provided, the patient was diagnosed with a longitudinal tear of the discoid medial meniscus (Fig. 2); the vertical tear extended through the entire thickness of the medial meniscus, and the inner fragment was displaced into the intercondylar notch. The probe is then removed, and an arthroscopic knife is carefully inserted through the standard anteromedial portal. The anterior side of the discoid medial meniscus is cut 7 mm from the periphery of the meniscus (Fig. 3). In the case example, we only needed to cut along the longitudinal tear to the anterior horn, as the tear shape was short and regular.

**Fig. 4:**
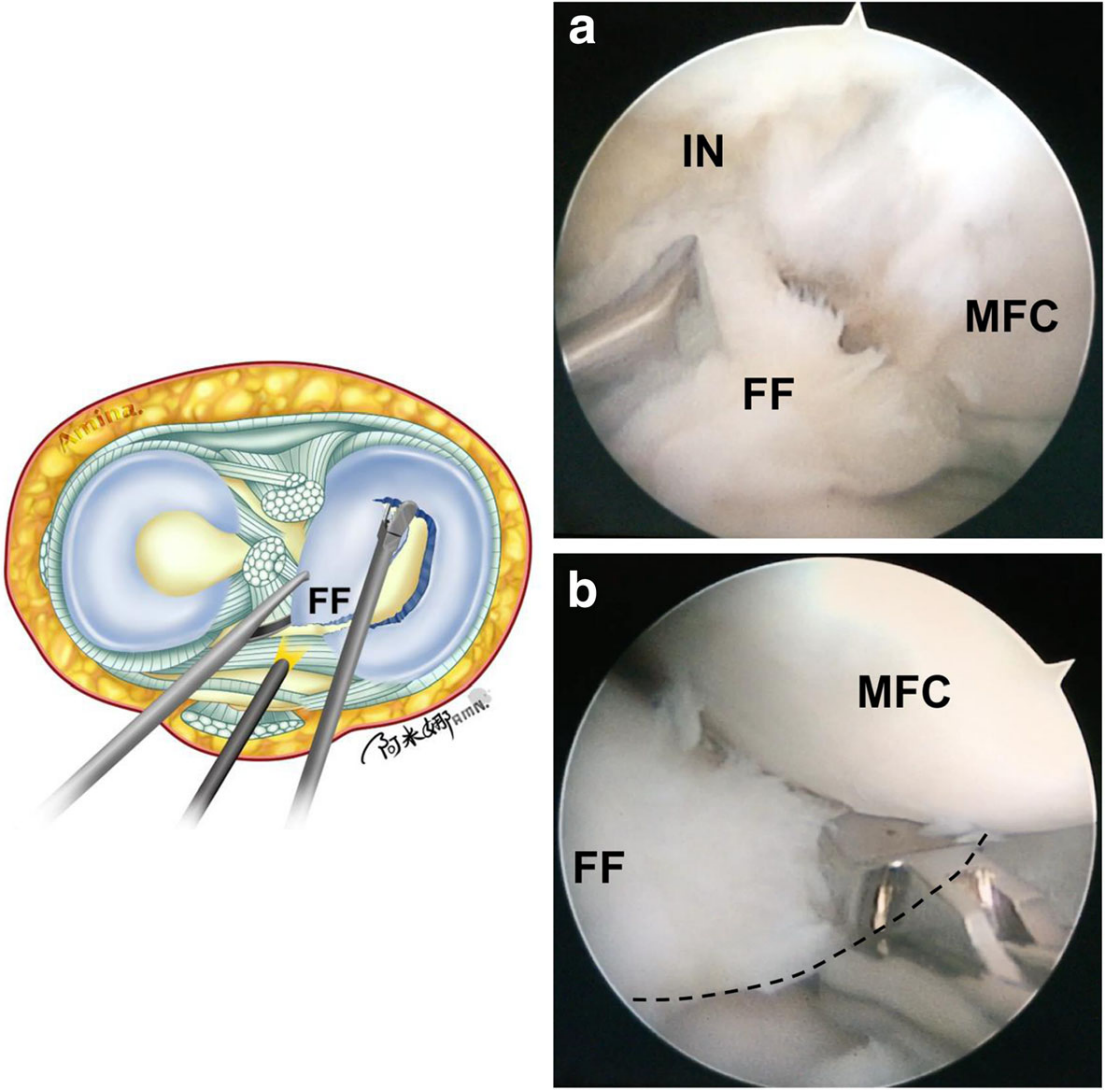
Illustration and intraoperative photographs showing the cutting of the damaged discoid medial meniscus. **a** A grasper was inserted through the high anterolateral portal. The anterior portion of the free discoid meniscus fragment was pulled in the anterolateral direction with tension. **b** A curve-shaped cut (black dotted line) was made along the longitudinal tear to the posterior horn using basket forceps through the standard anteromedial portal. IN intercondylar notch, MFC medial femoral condylar, FF free fragment

Second, a grasper is inserted through the high anterolateral portal. The arthroscope is fixed by an assistant to provide a view of the posterior horn of the meniscus. The anterior portion of the free discoid meniscal fragment is pulled in the anterolateral direction with tension (Fig. 4a). Then, a curve-shaped cut is made along the longitudinal tear to the posterior horn using basket forceps through the standard anteromedial portal (Fig. 4b). The posterior side of the discoid medial meniscus is cut 7 mm from the periphery of the meniscus.

**Fig. 5:**
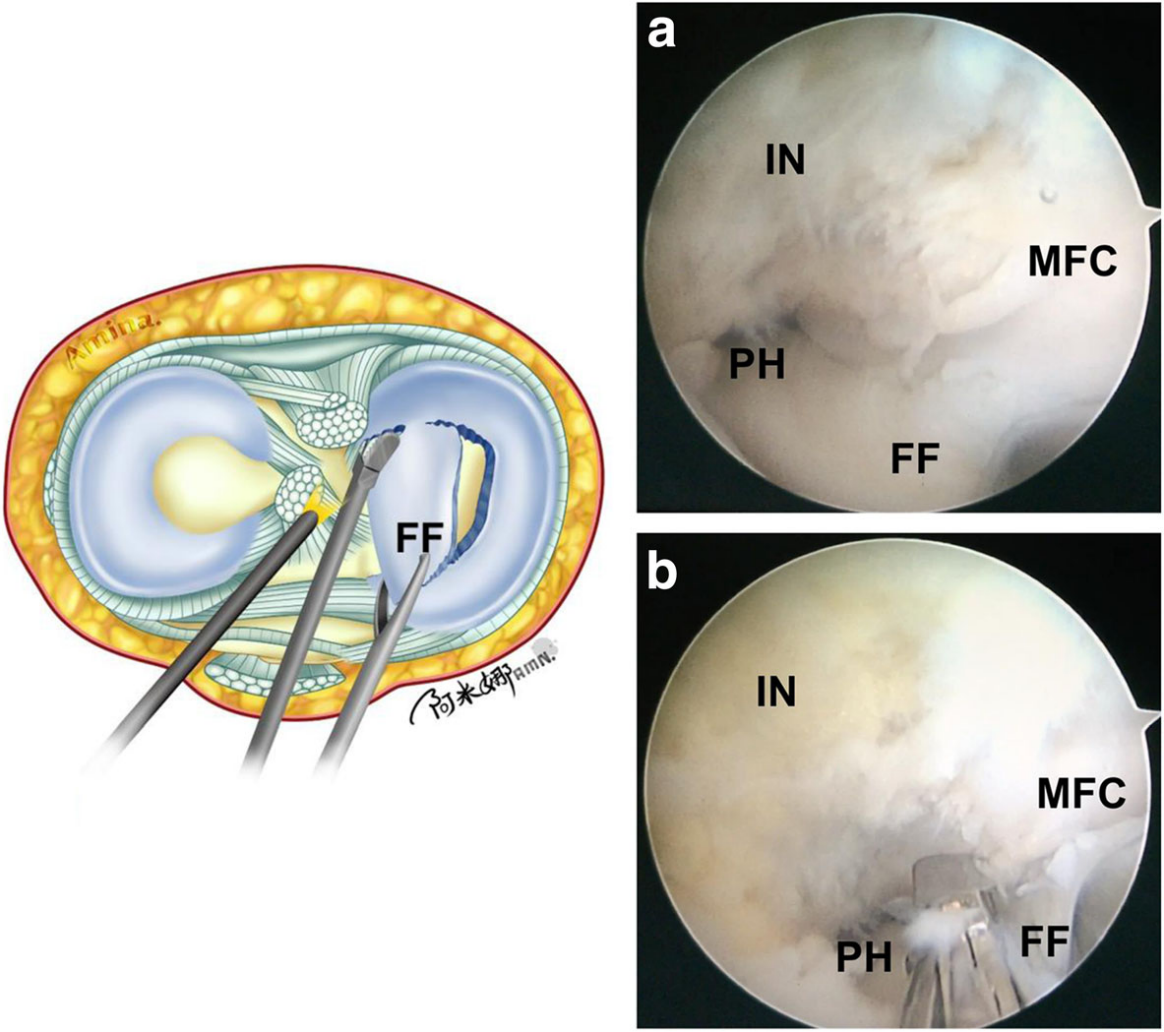
Illustration and intraoperative photographs showing the removal of the posterior portion of the medial meniscus. **a** A grasper was moved into the standard anteromedial portal. The anterior portion of the free discoid meniscus was pulled in the anteromedial direction to expose the posterior horn clearly. **b** The posterior side of the discoid meniscus was cut 7 mm from the periphery of the meniscus with straight scissors or basket forceps through the central transpatellar tendon portal. IN intercondylar notch, MFC medial femoral condylar, FF free fragment, PH posterior horn

Third, the arthroscope is placed through the high anterolateral portal and fixed by an assistant. A grasper is moved into the standard anteromedial portal. The anterior portion of the free discoid meniscus is pulled in the anteromedial direction to expose the posterior horn clearly (Fig. 5a). Pulling the fragment under tension makes it easier to cut the posterior side of the discoid meniscus. Then, the posterior side of the discoid meniscus is cut 7 mm from the periphery of the meniscus with straight scissors or basket forceps through the central transpatellar tendon portal. The central portion of the discoid meniscus is separated (Fig. 5b).

**Fig. 6:**
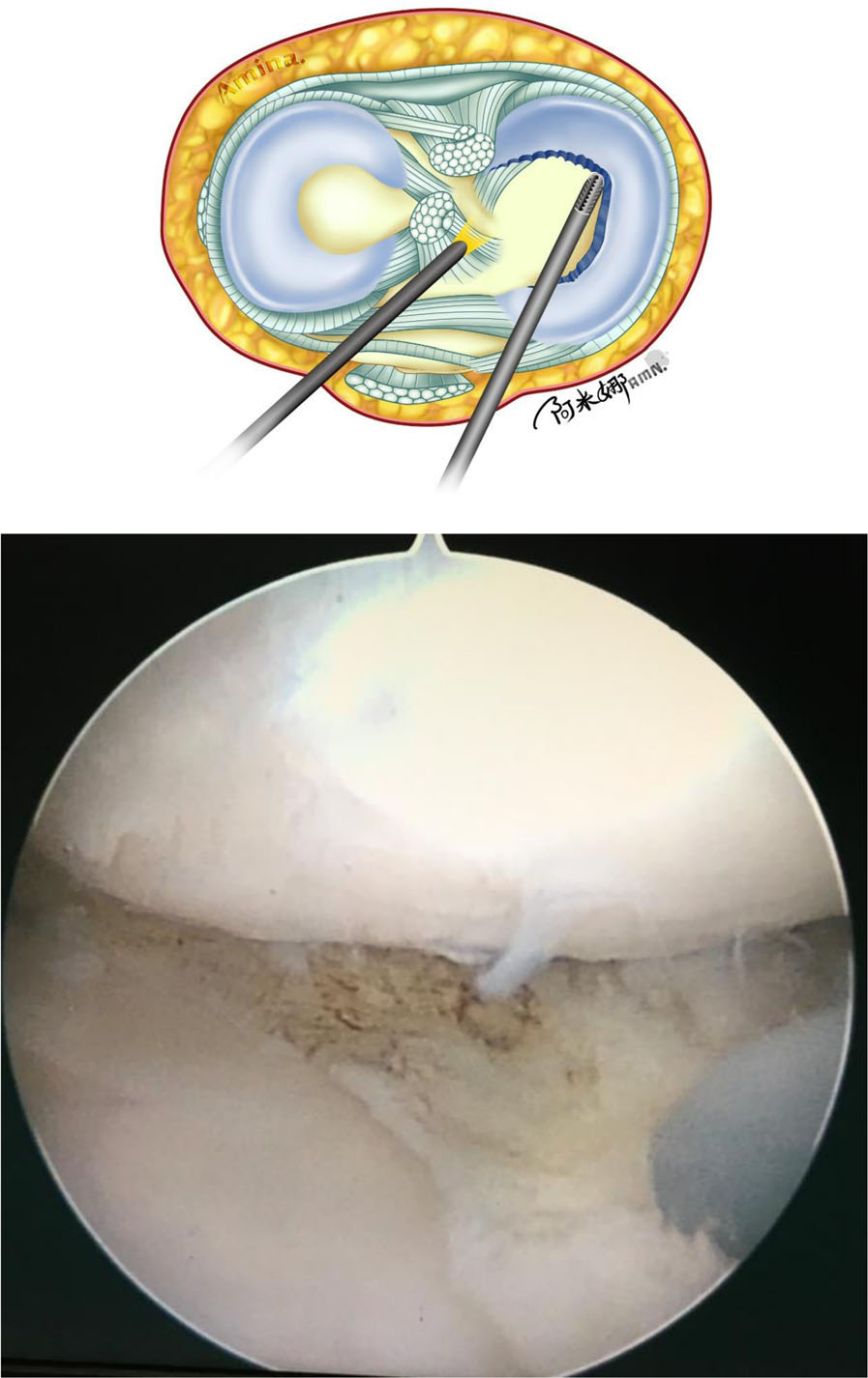
Illustration and intraoperative photograph showing the final steps of the removal of the medial meniscus. A motorized shaver and a radiofrequency instrument were inserted through the standard anteromedial portal respectively and used to smooth the inner rim of the remaining meniscus

Finally, after extraction of the central portion of the discoid meniscus in one piece through the standard anteromedial portal using a grasper, a motorized shaver and a radiofrequency instrument are inserted through the standard anteromedial portal respectively and used to smooth the inner rim of the remaining meniscus (Fig. 6).

## Discussion

Several techniques for the treatment of discoid meniscus have been described [6–8]. However, these excellent techniques are used for repairing discoid lateral meniscal tears. In contrast, only few reports have described the treatment of discoid medial meniscus. Anatomic studies report that the medial meniscus is C-shaped, in contrast to the uniform circular shape of the lateral meniscus [9, 10]. Furthermore, the medial meniscus has a posterior horn that is markedly wider than the anterior horn, and the anteroposterior dimension of the medial meniscus is larger than that of the lateral meniscus. So when the posterior portion of the discoid medial meniscus is cut, we cannot achieve clear visualization using the techniques for discoid lateral meniscal tear.

**Fig. 7:**
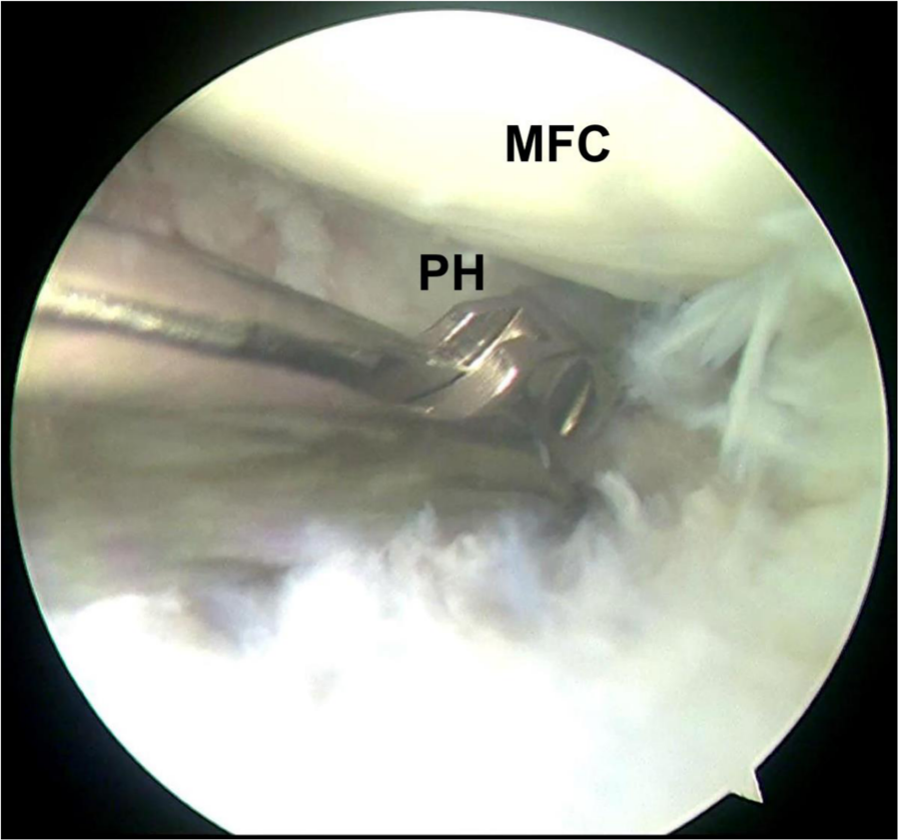
Using a two standard portals technique: anterolateral portal (viewing portal) and anteromedial portal (working portal), to cut the posterior portion of the discoid medial meniscus (left knee). The posterior horn cannot be viewed clearly viewing through anterolateral portal without being pulled forward. So the basket forceps is inserted deeper to cut the posterior portion. MFC medial femoral condylar, PH posterior horn

Kim et al. [11] described a technique for arthroscopic excision of the discoid medial meniscus in one piece using three portals. The posterior side of the medial meniscus is cut using a straight scissor punch through the high anterolateral portal. However, because of the obstruction of the ACL and the tibial intercondylar eminence and the narrow medial compartment, the working space is confined. Hence, it is difficult to cut the posterior side of the discoid medial meniscus through the high anterolateral portal, which may increase the risk of iatrogenic injury of the ACL and cartilage. In other previous cases shown in Fig. 7, we used a two standard portals technique: anterolateral portal and anteromedial portal, to treat this pathology. To cut the posterior portion, an assistant should provide a lager valgus stress to extend the medial compartment and the surgeon should insert the basket forceps deeper to cut using the anteromedial portal viewing though the anterolateral portal. This may increase the risk of iatrogenic injury of the MCL (medial collateral ligament) and leave some tearing portion that cannot be cut in the blind zone. To solve these problems, we propose a modified technique that uses two steps to cut the posterior portion of the discoid medial meniscus. Pulling the fragment forward under tension combined with viewing through the central transpatellar tendon portal and the high anterolateral portal can achieve a closer visualization of the posterior portion. This can provide a larger working place for basket forceps and eliminate the blind zone of the posterior portion of the discoid medial meniscus compared with viewing through the anteromedial portal. Furthermore, cutting through the standard anteromedial portal and the central transpatellar tendon portal in two separate steps can avoid the obstruction caused by the ACL and the tibial intercondylar eminence and does not need a larger valgus stress and a deeper insertion anymore. Hence, our technique is easier, quicker, and more accurate than the previously described technique and decreases the risk of ACL, MCL, and cartilage injury. Because of the excellent visualization of the posterior portion achieved by three improved portals, more than 20 min of operating time is rarely required in our recent experience. In addition, the curve-shaped cut in our technique can restore the anatomic morphology of the inner posterior rim of the medial meniscus.

Previously, symptomatic discoid meniscus was treated via total meniscectomy [12, 13]. However, some authors reported that total meniscectomy led to progressive osteoarthritis [14, 15]. The meniscus plays an important role in knee stability, load transmission, and articular cartilage nutrition. So to maintain the function of the meniscus, partial meniscectomy is recommended for symptomatic discoid medial meniscus. The width of the rim of the remaining meniscus is dependent on the type of meniscus involved and the shape and extent of the meniscal tear. Many authors have reported that the width of the remaining meniscal rim should be between 4 and 8 mm [6, 16, 17]. We retained a rim that was 7 mm wide to achieve a stable rim, with a final rim of about 5 mm after motorized shaver smoothing. A number of foreign bodies may be produced during the cutting procedure. To reduce this problem, Hayashi et al. [18] and Vandermeer and Cunningham [16] suggested the partial excision technique for removing the discoid meniscus in one piece; however, they did not describe the technique in detail. Hence, we have introduced this improved arthroscopic technique for partial meniscectomy in one piece that we have successfully used in six cases.

Excellent results have been obtained from these six patients with symptomatic medial discoid meniscus using this technique from January 2010 to January 2015. No recurrent symptoms were found. Patient demographics are shown in Table 1. International Knee Documentation Committee (IKDC) score, Lysholm score, and visual analog scale (VAS) of pain were improved at 2-year final follow-up (Table 2).

**Table 1:** Patient demographics

Demographic data	Value
No. of cases	6
Gender (F/M)	2/4
Side (L/R)	3/3
Age at operation (year)	25.7 ± 9.8
Follow-up periods (month)	25.2 ± 1.2

**Table 2:** Clinical results of patients

	Preoperative	Postoperative	*P* value
IKDC	38.1 ± 6.0	83.3 ± 5.7	< .001
Lysholm	48.3 ± 3.9	84.5 ± 3.9	< .001
VAS	7.5 ± 1.0	2.5 ± 1.4	< .001

*IKDC* International Knee Documentation Committee, *VAS* visual analog scale

Our report has two limitations. First, although we achieved satisfactory clinical outcomes, to create the central transpatellar tendon portal, a vertical incision must be made through the patellar tendon, which may cause short-term postoperative anterior knee pain. We found this complication in four of the six cases. The duration of the anterior knee pain was about 1 month. The pain was completely gone at 2 months postoperatively. Second, as discoid medial meniscus is an extremely rare abnormality of the knee, the number of patients was limited. In addition, a potential weakness in the present study was the short-term follow-up period, and we also did not evaluate postoperative radiology changes of the patients. So we will add a larger number of cases and radiology outcomes and prolong the follow-up period in further study.

## Conclusion

This improved arthroscopic one-piece excision technique for the treatment of symptomatic discoid medial meniscus enables the posterior part of the meniscus to be cut satisfactorily. Compared with previous techniques, this novel technique is associated with less formation of foreign bodies; less damage to the ACL, MCL, and cartilage; and a shorter procedural time. More cases and additional follow-up results are needed to verify the overall effect of this technique.

## Abbreviations

ACL: Anterior cruciate ligament
IKDC: International Knee Documentation Committee
MCL: Medial collateral ligament
VAS: Visual analog scale
